# Supplementary material for: De novo genome assembly depicts the immune genomic characteristics of cattle
Source: Nat Commun. 2023 Oct 19;14:6601. doi: 10.1038/s41467-023-42161-1 (PMC10587341; doi:10.1038/s41467-023-42161-1)
Supplement: Supplementary file 1 — Supplementary Information [file 41467_2023_42161_MOESM1_ESM.pdf]

Supplementary Information for

De novo Genome Assembly Depicts the Immune Genomic Characteristics of Cattle

Ting-Ting Li[1†], Tian Xia[1†], Jia-Qi Wu[1†], Hao Hong[1], Zhao-Lin Sun[2], Ming Wang[3,4], Fang-Rong Ding[3], Jing Wang[2], Shuai Jiang[1], Jin Li[1], Jie Pan[1], Guang Yang[2], Jian-Nan Feng[2], Yun-Ping Dai[3], Xue-Min Zhang[1,5], Tao Zhou[1]* & Tao Li[1,5]*

[1]Nanhu Laboratory, National Center of Biomedical Analysis, 27 Tai-Ping Road, Beijing 100850, China.

[2]State Key Laboratory of Toxicology and Medical Countermeasures, Beijing Institute of Pharmacology and Toxicology, Beijing 100850, China.

[3]State Key Laboratories for Agrobiotechnology, College of Biological Sciences, China Agricultural University, No.2 Yuanmingyuan Xilu, Beijing 100193, China.

[4]College of Animal Science and Technology, China Agricultural University, No.2 Yuanmingyuan Xilu, Beijing 100193, China.

[5]School of Basic Medical Sciences, Fudan University, Shanghai 200032, China.

[†]These authors contributed equally: Ting-Ting Li, Tian Xia, Jia-Qi Wu

*Corresponding authors. Email: tli@ncba.ac.cn (T.L.), tzhou@ncba.ac.cn (T.Z.)

This supplementary file includes：Supplementary Figures 1-16

Supplementary Fig. 1

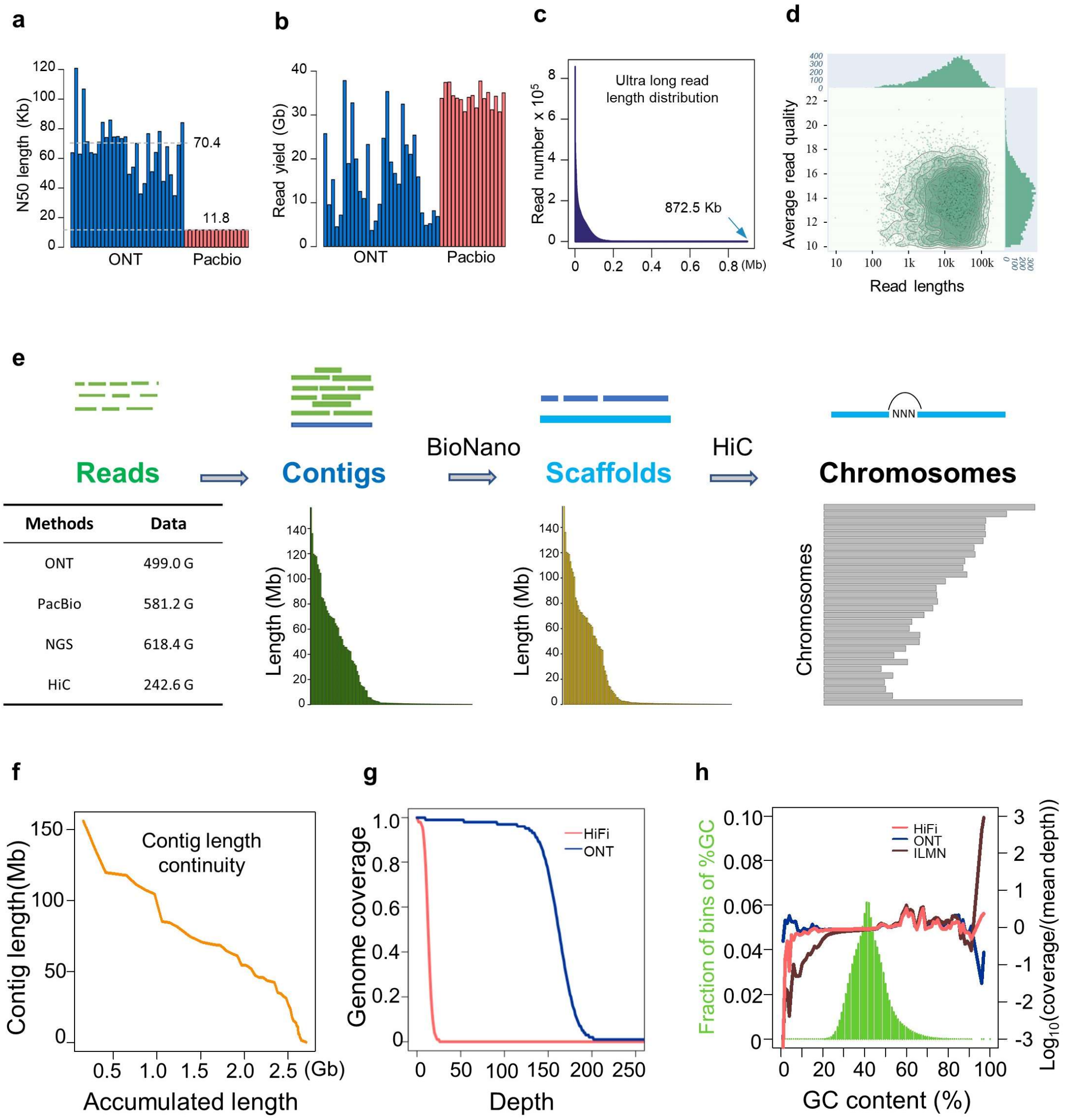

Supplementary Fig. 1. Data summary of cattle genomic assembly.

(a) Read length distributions of ONT ultra-long and PacBio HiFi methods. Each bar indicates the N50 read length of a separate sequencing flow cell.

(b) Read yield distributions of ONT ultra-long and PacBio HiFi methods. Each bar indicates a separate sequencing flow cell.

(c) Length distribution of the total ONT ultra-long reads.

(d) Nanoplot of raw ONT ultra-long reads.

(e) Flow chart of the genome assembly pipeline.

(f) Continuity of ultra-long reads assembled contigs.

(g) Read coverage of NCBA_BosT1.0 by ONT ultra-long reads and HiFi reads.

(h) Influence of GC content on genome coverage of three sequencing methods.

Supplementary Fig. 2

a

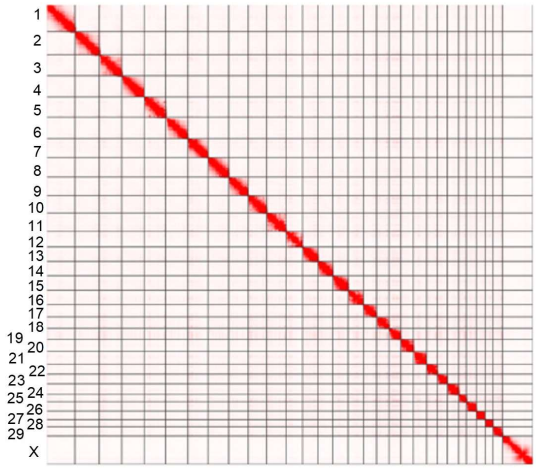

b

Base-level accuracy statistic of Genome

Type	Depth >= 1x	Depth >= 5x	Depth >= 10x
Hetero SNP	4,716,965	4,714,858	4,698,447
Hetero Indel	486,094	480,010	467,323
Homo SNP	15,992	10,813	7,428
Error rate by Homo SNP(%)	0.000591	0.000399	0.000274
Homo Indel	14,437	10,738	5,298
Error rate by Homo Indel(%)	0.000533	0.000397	0.000196
Error rate by Homo Variants(%)	0.001124	0.000796	0.000470
Accuracy genome(%)	99.998876	99.999204	99.999530

c

Assembly	Sequencing methods	Contig N50 (Mb)	No. of contigs	No. of Scaffold	Genome length (Gb)
NCBA_BosT1.0	ONT ultra long; PacBio	74.7	143	154	2.71
ARS-UCD1.2 (GigaScience, 2020)	PacBio	12	3077	2511	2.72
UOA_WB_1, Water buffalo (Nat Commun, 2019)	PacBio	18.8	953	509	2.65
ARS_Simm 1.0 (J Hered, 2021)	ONT	70.8	1374	1315	2.86

Supplementary Fig. 2. Correctness evaluation of the new assembly.

(a) Global 3D genome heatmaps of the new assembly by *in situ* Hi-C.

(b) base-level accuracy evaluation of the new assembly.

(c) Summary of recently assembled genomes related to bovine.

Supplementary Fig. 3

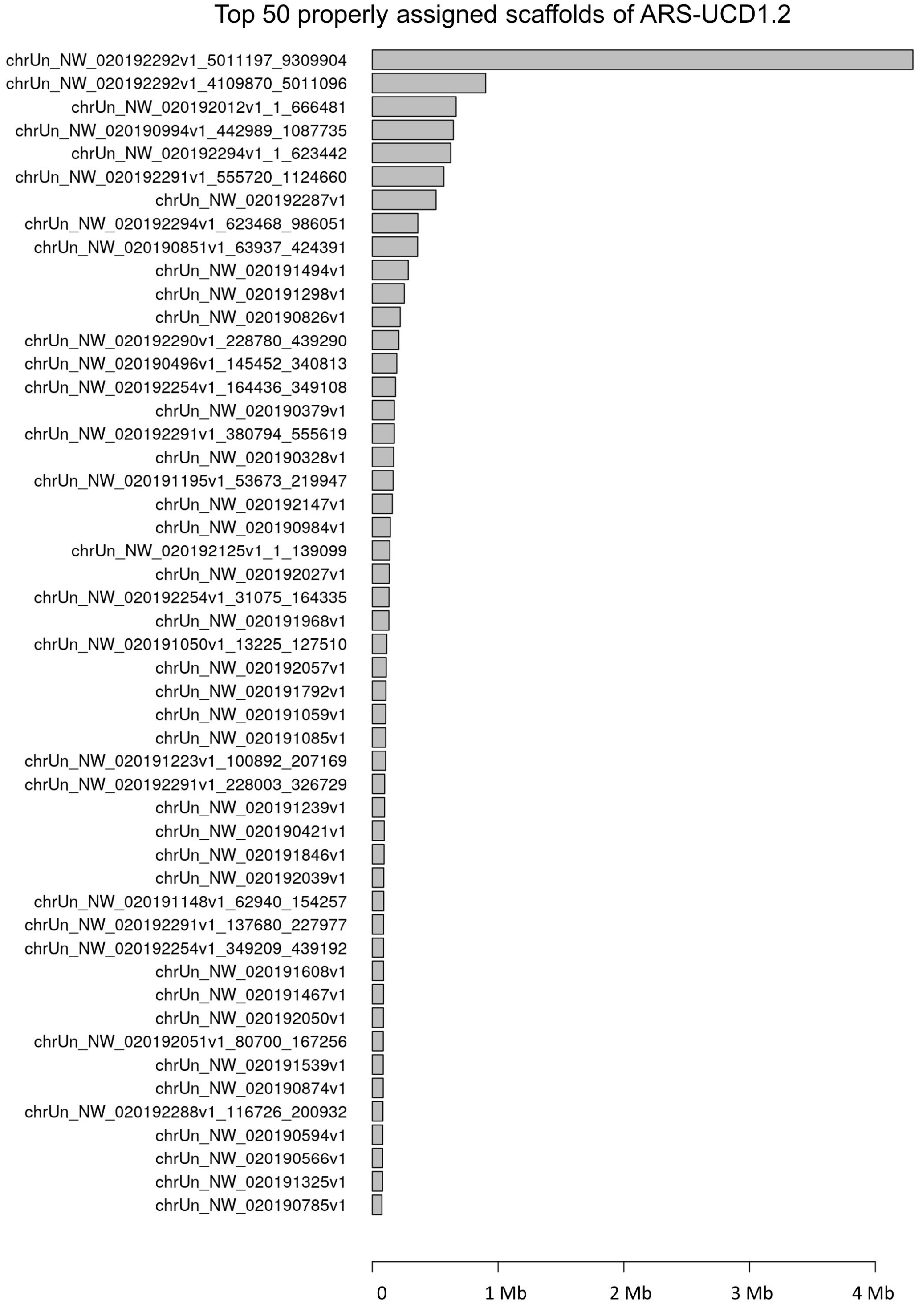

Supplementary Fig. 3. Properly placed scaffolds of ARS-UCD1.2 in NCBA_BosT1.0.

Top fifty scaffolds of ARS-UCD1.2 according to their sequence length were shown. Information of all properly placed scaffolds were stored in Table S2.

Supplementary Fig. 4

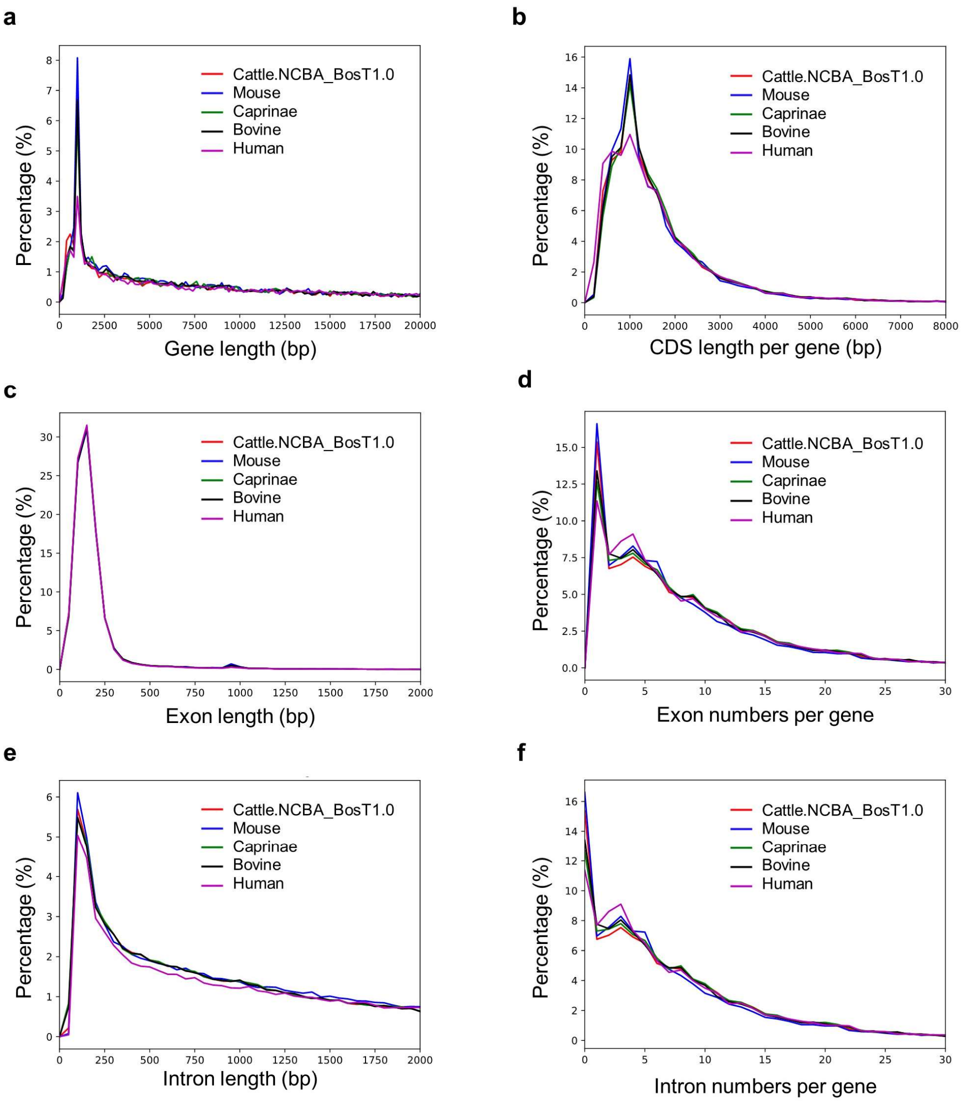

Supplementary Fig. 4. Gene length distributions of five species.

(a) Distribution of the gene lengths of five species.

(b) Distribution of the CDS lengths of five species.

(c) Distribution of the exon lengths of five species.

(d) Distribution of the exon numbers per gene of five species.

(e) Distribution of the intron lengths of five species.

(f) Distribution of the intron numbers per gene of five species.

Supplementary Fig. 5

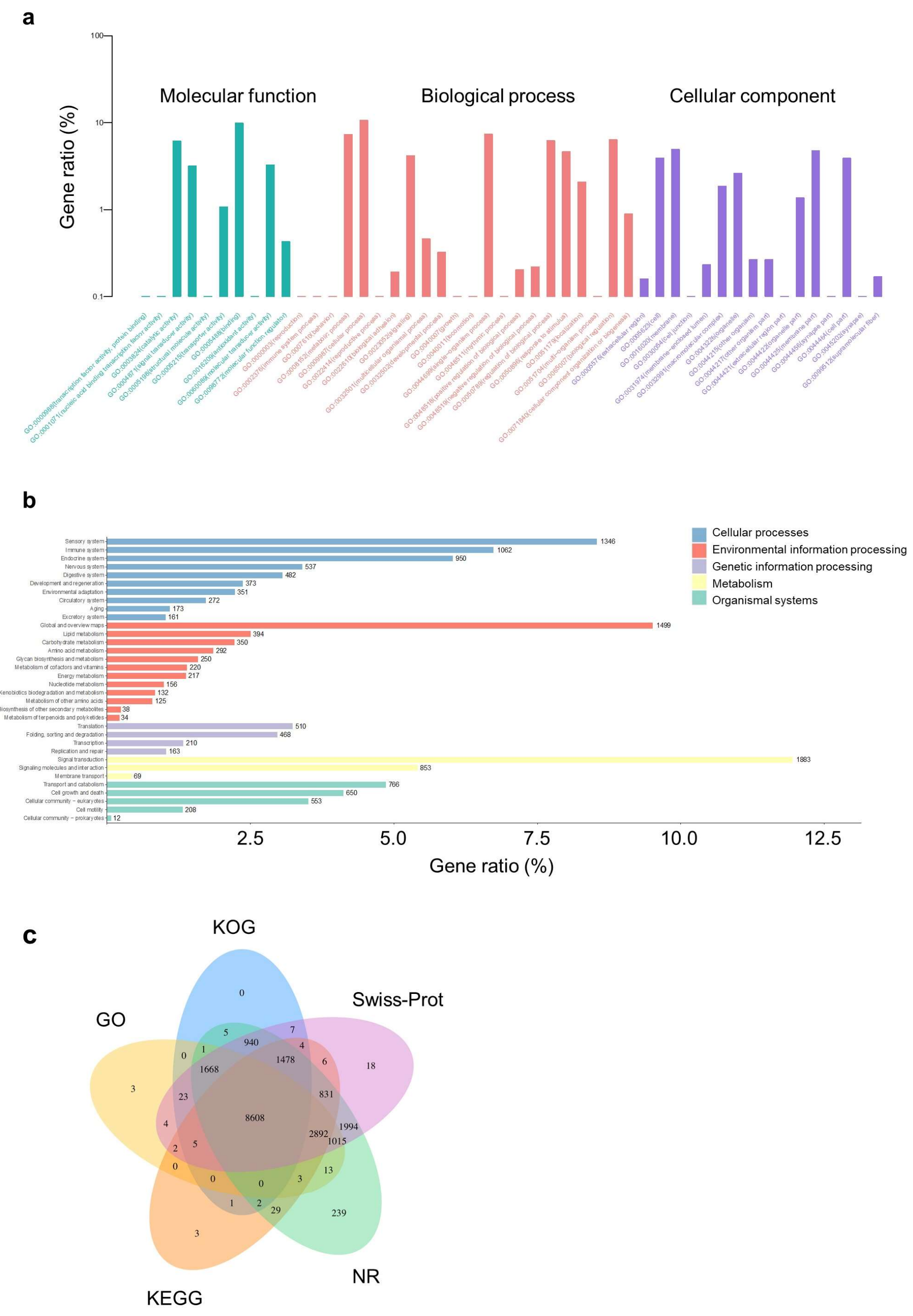

Supplementary Fig. 5. Functional annotation of predicted genes in NCBA_BosT1.0.

(a) GO annotation of predicted genes.

(b) KEGG Ortholog annotation of predicted genes.

(c) Venn diagram of genes annotated to KOG, GO, KEGG, NR and Swiss-Prot.

Supplementary Fig. 6

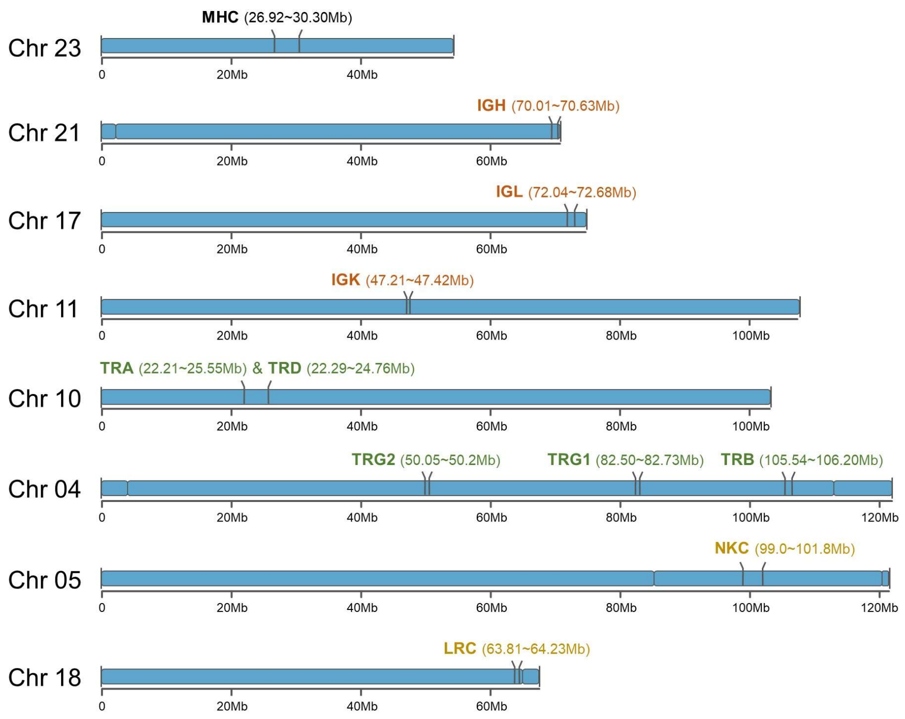

Supplementary Fig. 6. Immune gene loci in NCBA_BosT1.0 assembly.

The IG, TR, MHC genomic loci and NK receptor loci dispersed in eight chromosomes. The genomic coordinates for each locus were labeled and the gaps between contigs were annotated too. All immune loci were seamlessly assembled.

Supplementary Fig. 7

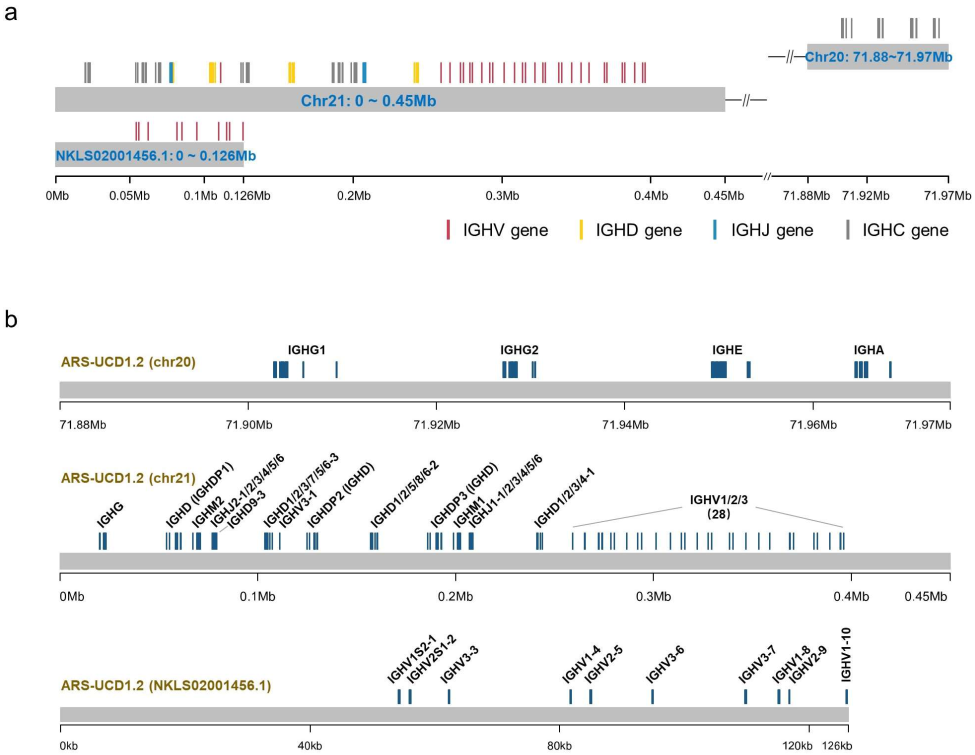

Supplementary Fig. 7. IGH loci in ARS-UCD1.2.

(a) Genomic organization of IGH loci in ARS-UCD1.2. The gene segments of V, D, J and C were labeled using different colors.

(b) Detailed diagrams of IGH gene structures and annotations in three regions, including chromosomes 20, 21 and unplaced scaffold NKLS02001456.1.

Supplementary Fig. 8

a

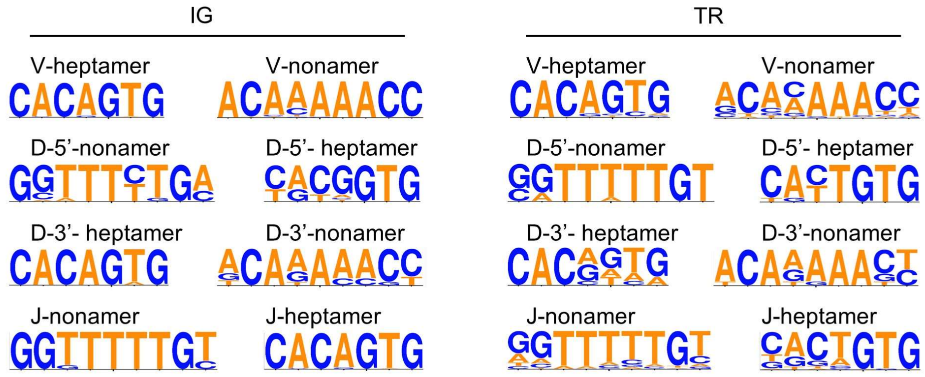

b

Functional gene	L-PART	Gene body	RS signal
V	• L-1 starts with ATG;<br>• Length of (L1 + L2) is 3N;<br>• Length of V-Intron < 500 nt;<br>• No stop codon;<br>• L-1 donor splice site is "GT";<br>• L-2 acceptor splice site is "AG"	• 1st-CYS in the 23rd AA, 2nd-CYS in the 104th AA, and TRP in the 41th AA (or a nearby position);<br>• No stop codon(except the last codon in CDR3 region); no frameshift;<br>• No long deletions in CDR1/2 region	• Canonical nonamer and heptamer with no more than 1 mismatch;<br>• The size variation of X-Spacer length less than 3
D	NA	• No requirement	• Canonical nonamer and heptamer with no more than 1 mismatch;<br>• The size variation of X-Spacer length less than 3
J	NA	• FGXG motif in light chain and WGXG motif in heavy chain,	• Canonical nonamer and heptamer with no more than 1 mismatch;<br>• The size variation of X-Spacer length less than 3
C	NA	• No stop codon and no frameshift	NA

Pseudogene	L-PART	Gene body	RS signal
V	• Stop codon or frameshift;<br>• No L-1 or L-2;<br>• No INIT codon;<br>• No L1 donor splice site;<br>• No L2 acceptor splice site	• Stop codon or frameshift;<br>• A part of V-region is missing	• No nonamer and/or heptamer
D	NA	NA	• No nonamer and/or heptamer
J	NA	NA	• No nonamer and/or heptamer
C	NA	• Stop codon or frameshift	NA

Open Reading Frame (ORF)	L-PART	Gene body	RS signal
V	• Noncanonical L1 donor splice site;<br>• Noncanonical L2 acceptor splice site;<br>• Unexpected V-Intron length	• Mutated 1st-CYS in the 23rd AA, mutated 2nd-CYS in the 104th AA, or mutated TRP in the 41th AA (or a nearby position);<br>• Deletion of more than 3AA in V-region	• Noncanonical nonamer and/or heptamer;<br>• Unexpected X-Spacer length;
D	NA	NA	• Noncanonical nonamer and/or heptamer;<br>• Unexpected X-Spacer length;
J	NA	• Mutated FGXG motif in light chain or mutated WGXG motif in heavy chain,	• Noncanonical nonamer and/or heptamer;<br>• Unexpected X-Spacer length;
C	NA	NA	NA

Supplementary Fig. 8. Criteria for IG/TR gene structures and functionality annotation.

(a) Sequence conservation logos were created with recombination signal sequences of all functional cattle genes from IMGT by WebLogo software.

(b) Criteria for determining the functionality of an IG/TR gene. The functionality of an IG/TR gene is defined as functional (F), Open Reading Frame (ORF) or Pseudogene (P) based on the sequence analysis.

Supplementary Fig. 9

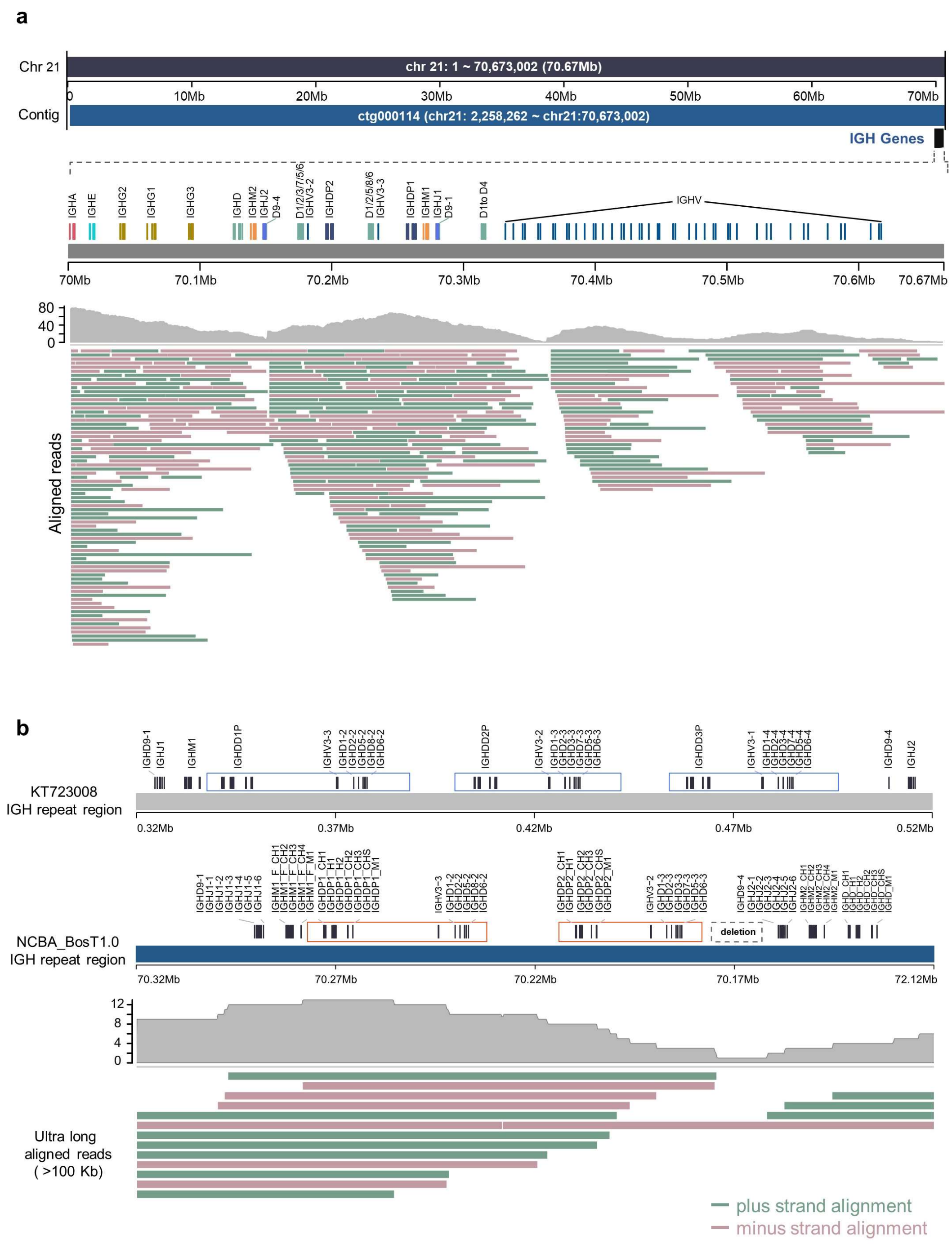

Supplementary Fig. 9. Ultra-long reads coverage of IGH locus.

(a) Global view of the IGH locus covered by ONT ultra-long reads.

(b) Enlarged tandem repeat regions of IGH locus. The repeated regions were represented as blue and red rectangles in KT723008 and NCBA_BosT1.0, respectively. In newly assembled NCBA_BosT1.0, there is a deletion of tandem repeat [IGHDP-IGHV3-(IGHDv)5/6] in IGH locus, which was labeled as gray rectangle.

Supplementary Fig. 10

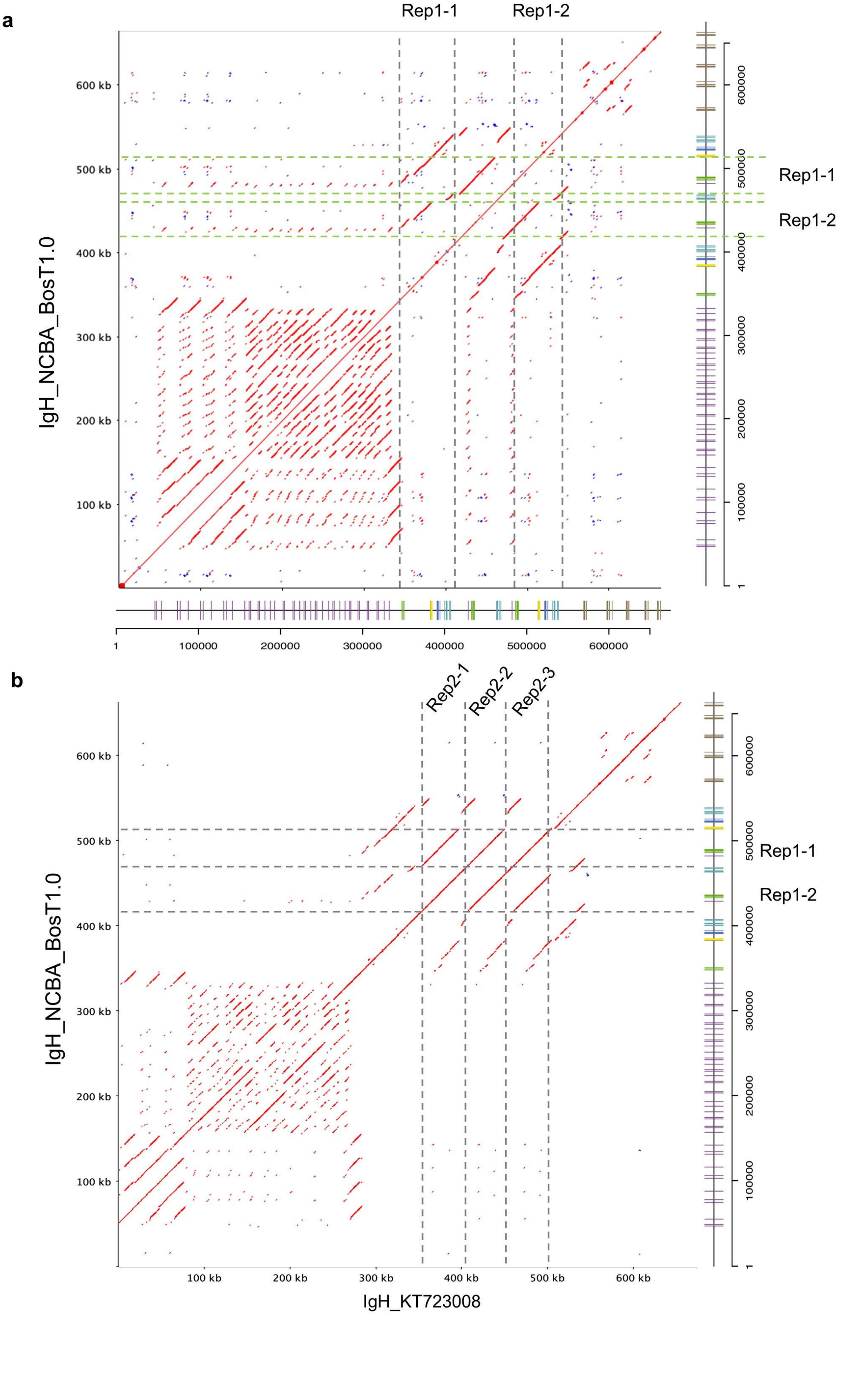

Supplementary Fig. 10. Dot plots between two IGH genomic sequences.

(a) Dot plot of the IGH genomic sequence in NCBA_BosT1.0 assembly. The repeated regions were labeled as rep1-1 and rep1-2.

(b) Pairwise alignment between IGH in NCBA_BosT1.0 and previously reported IGH sequence (KT723008). The three tandem repeats in KT723008 were labeled as rep2-1, rep2-2 and rep2-3.

Supplementary Fig. 11

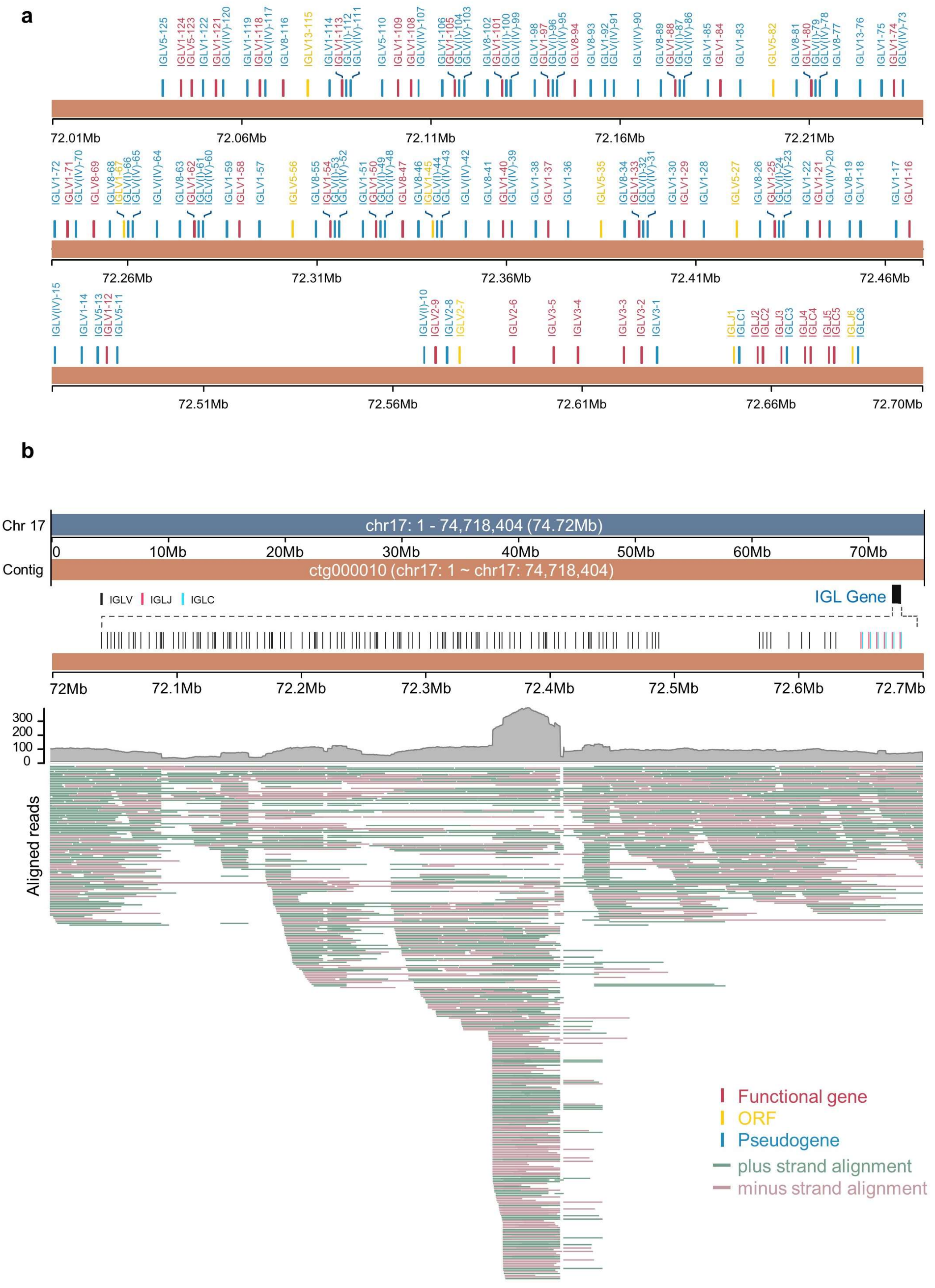

Supplementary Fig. 11. Detailed annotation map of IGL locus.

(a) Elaborate gene structures of IGL locus in NCBA_BosT1.0 assembly.

(b) Read coverage by ONT ultra-long reads in the IGL genomic region.

Supplementary Fig. 12

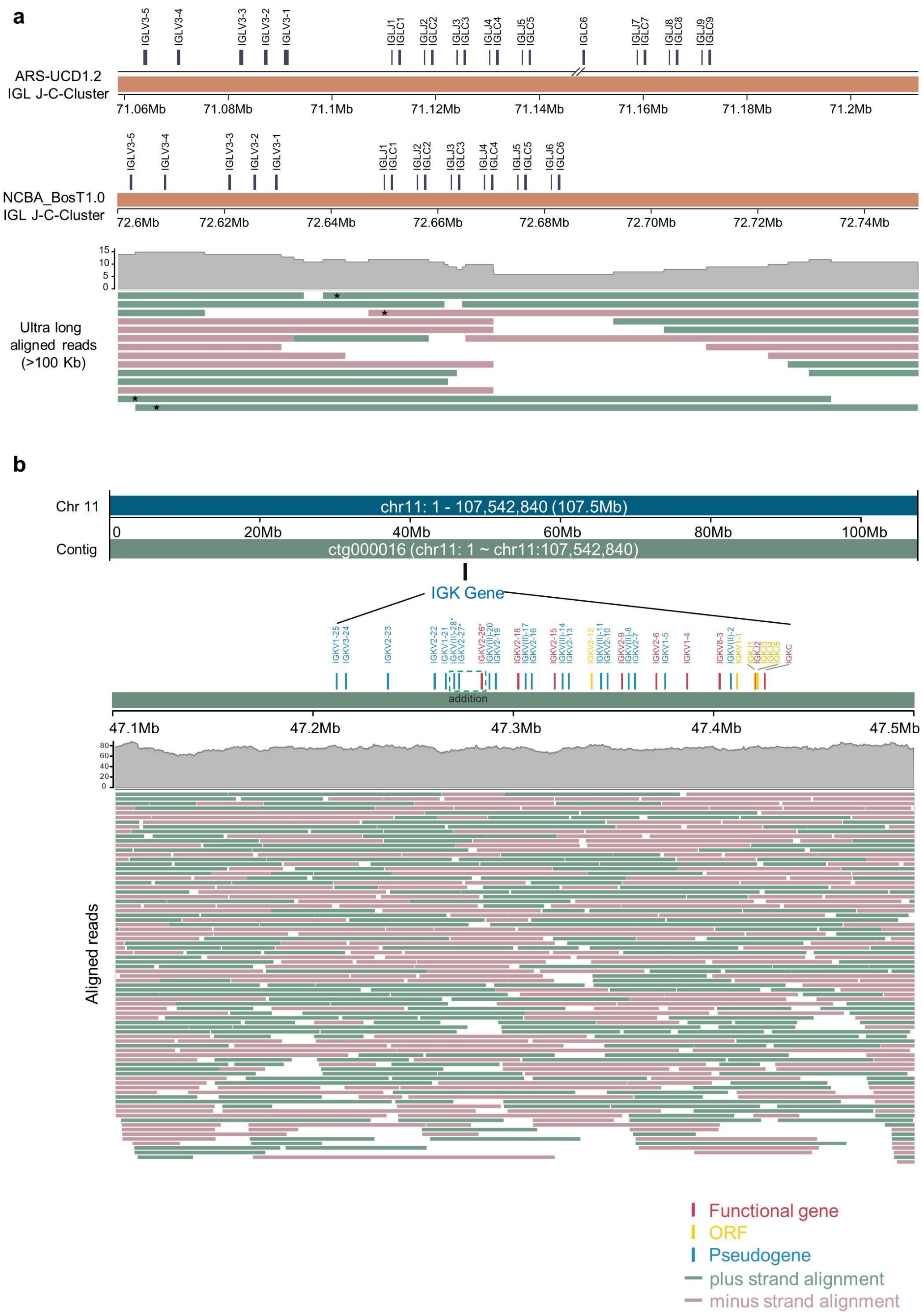

Supplementary Fig. 12. Enlarged alignment map of IGL J-C cluster region and annotation map of IGK locus.

(a) ONT ultra-long reads that longer than 100 Kb were aligned back to the IGL locus, and four separate ONT ultra-long reads that span over the entire IGL J-C cluster region were labeled with asterisk.

(b) Genomic coordinate and organization of IGK locus were depicted. The newly identified IGKV genes in NCBA_BosT1.0, compared to ARS-UCD1.2, have been assigned provisional names while retaining the established IMGT nomenclature. And the names of these genes were marked with asterisk in the figure. ONT ultra-long reads that longer than 100 Kb and the mapping to the genomic region were drawn.

Supplementary Fig. 13

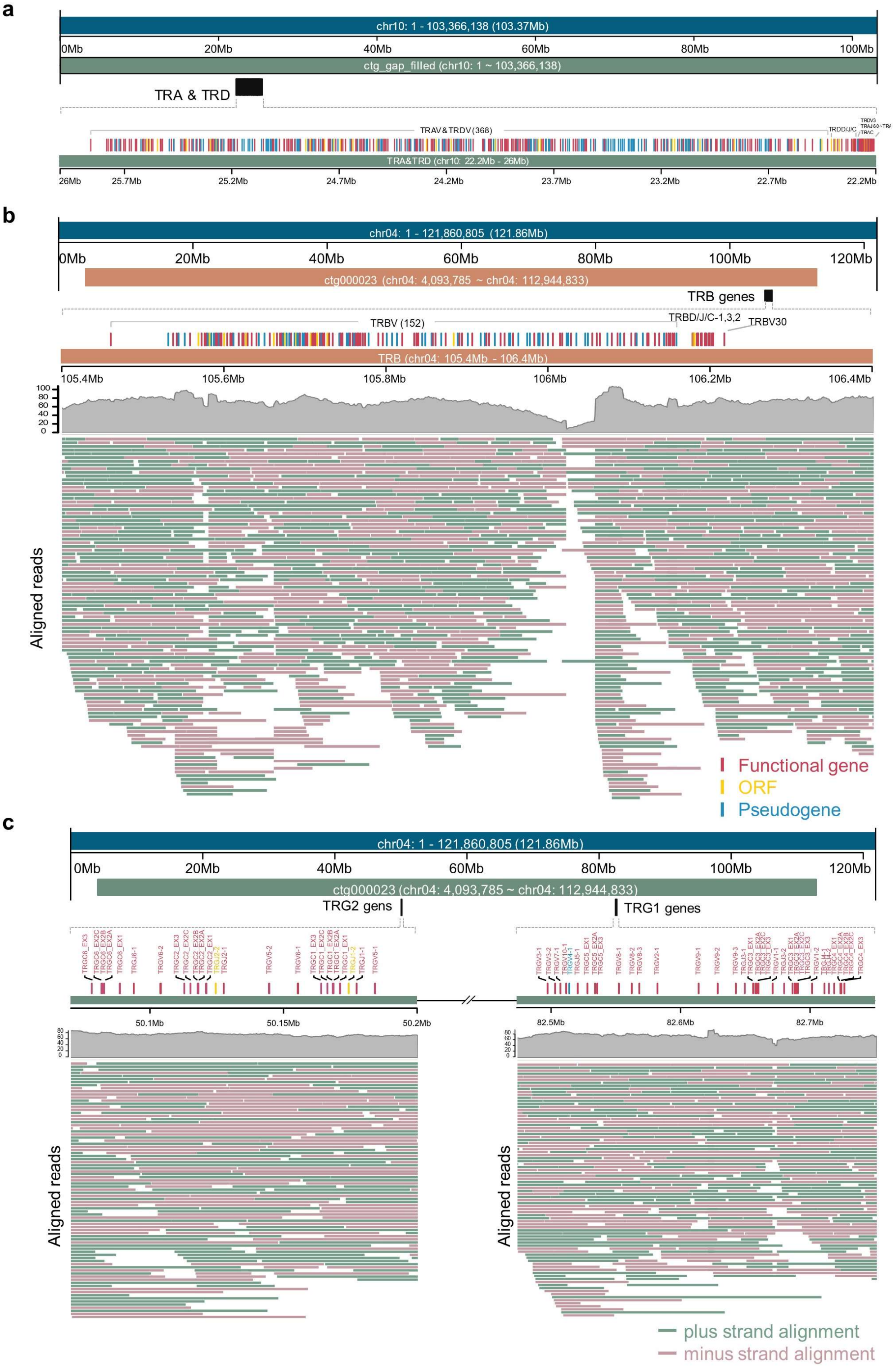

Supplementary Fig. 13. Global genetic and alignment map of the TR loci.

(a) Genomic coordinate and organization of TRA/TRD loci were annotated. The remaining two gaps within the TRA/D V gene region were depicted too.

(b) Genetic map of TRB locus in chromosome 4. TRB locus resides within contig23 and ONT ultra-long reads that mapped to the genomic region were drawn.

(c) Genetic map of TRG locus in chromosome 4. TRG contains two separate gene clusters: TRG1 and TRG2, that are 32 Mb distant away from each other. ONT ultra-long reads that mapped to the genomic region were drawn.

Supplementary Fig. 14

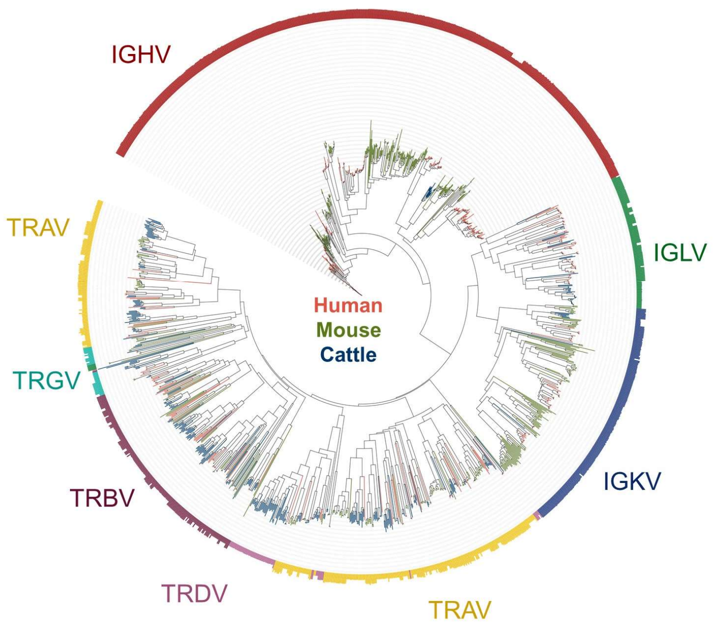

Supplementary Fig. 14. Phylogenetic analysis of the V genes.

Phylogenetic tree of all functional IG and TR V genes. V genes of human, mouse and cattle were merged together and clustered well according to their biological classes.

Supplementary Fig. 15

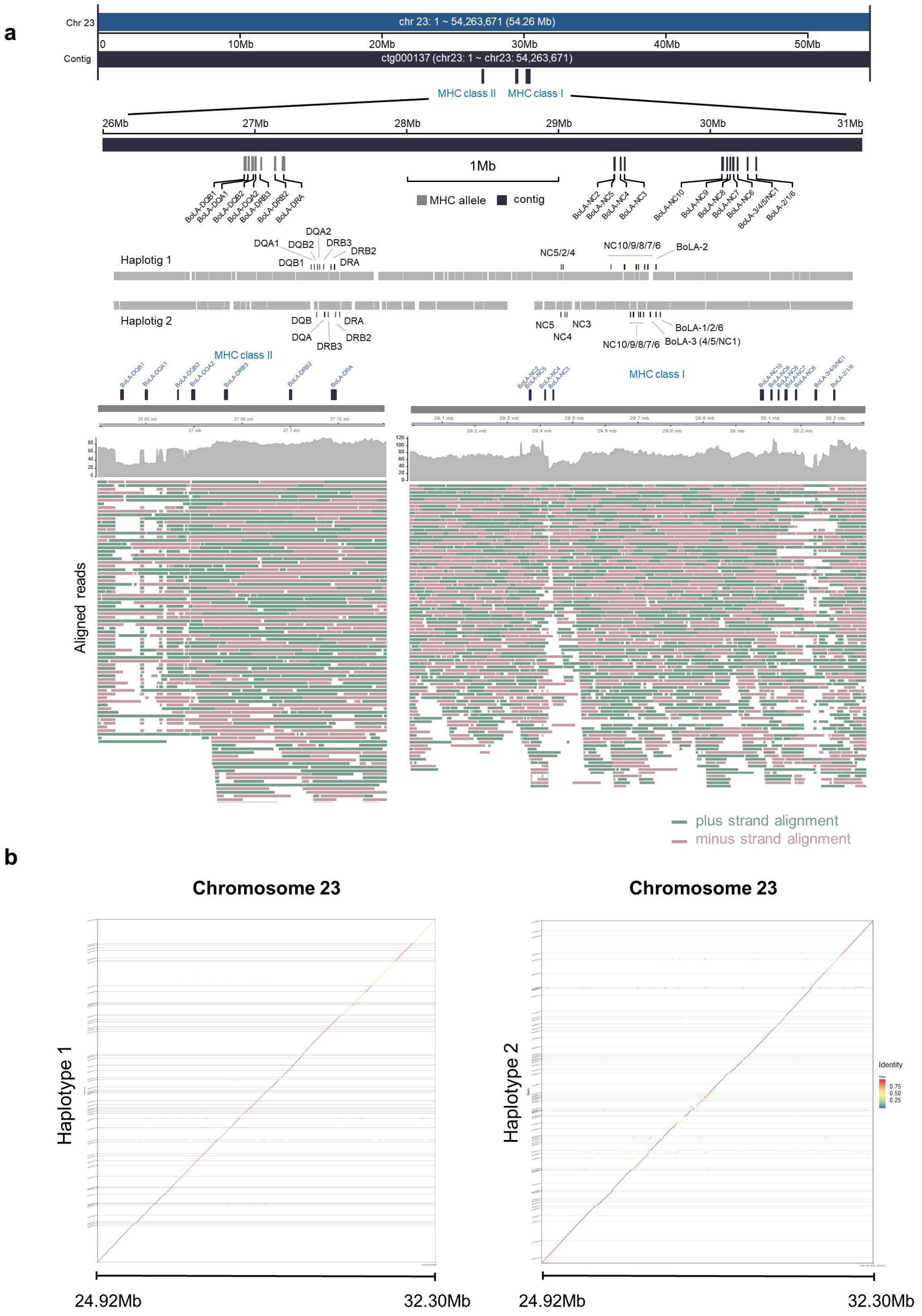

Supplementary Fig. 15. Genomic assembly and haplotyping of MHC locus.

(a) Genomic coordinate and annotation of MHC I and MHC II. ONT ultra-long reads that mapped to the genomic region were drawn.

(b) Sequence alignments between two haplotigs and the MHC genomic region. Colors indicate the sequence identity of the alignments.

Supplementary Fig. 16

a

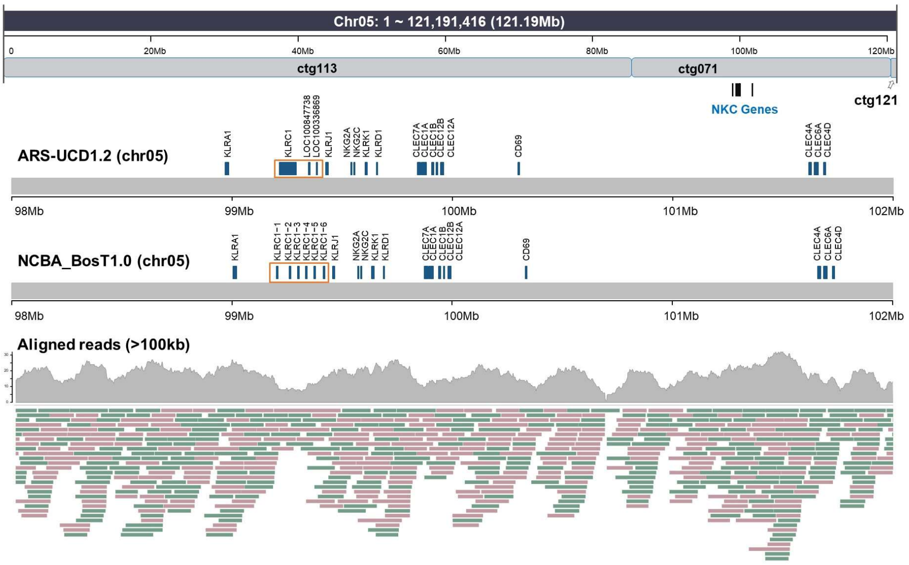

b

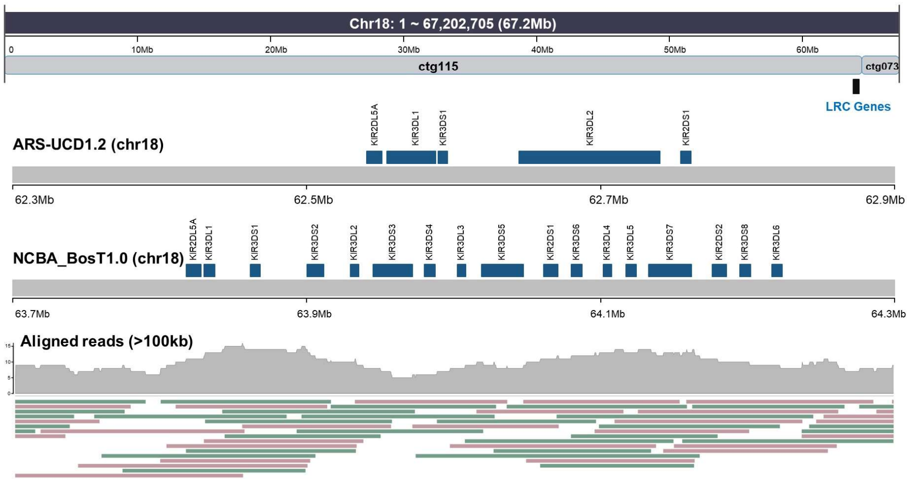

Supplementary Fig. 16. Global genetic maps of the NK receptor loci.

(a) Genomic organization and detailed gene annotation of NKC loci in ARS-UCD1.2 and NCBA_BosT1.0.

(b) Genomic organization and detailed gene annotation of LRC loci in ARS-UCD1.2 and NCBA_BosT1.0. ONT ultra-long reads that longer than 100 Kb and the mapping to the genomic region were drawn. The differences between the two genomes were labeled as orange rectangles.
