# Supplementary material for: De novo genome assembly depicts the immune genomic characteristics of cattle
Source: Nat Commun. 2023 Oct 19;14:6601. doi: 10.1038/s41467-023-42161-1 (PMC10587341; doi:10.1038/s41467-023-42161-1)
Supplement: Supplementary file 3 — Description of Additional Supplementary Files [file 41467_2023_42161_MOESM3_ESM.pdf]

Description of Additional Supplementary Files

File name: Supplementary Data 1

Description: Statistics of raw sequencing data

File name: Supplementary Data 2

Description: Contig length and their genomic coordinates

File name: Supplementary Data 3

Description: Statistics of the NCBA_BosT1.0 assembly

File name: Supplementary Data 4

Description: Genome coverage by ONT ultra-long reads, PacBio HiFi reads, and Illumina reads

File name: Supplementary Data 5

Description: Contig coordinates for each chromosome

File name: Supplementary Data 6

Description: Filled gaps of ARS-UCD1.2 by the NCBA_BosT1.0 assembly

File name: Supplementary Data 7

Description: Genomic coordinates of placed scaffolds of ARS-UCD1.2 in the NCBA_BosT1.0

File name: Supplementary Data 8

Description: Genomic annotations of the NCBA_BosT1.0

File name: Supplementary Data 9

Description: Gene statistics of cattle for each immune locus in the IMGT database

File name: Supplementary Data 10

Description: Contig length of MHC haplotypes
