# Supplementary material for: De novo genome assembly depicts the immune genomic characteristics of cattle
Source: Nat Commun. 2023 Oct 19;14:6601. doi: 10.1038/s41467-023-42161-1 (PMC10587341; doi:10.1038/s41467-023-42161-1)
Supplement: Supplementary file 5 — Reporting Summary [file 41467_2023_42161_MOESM5_ESM.pdf]

nature portfolio

Corresponding author(s): Tao Li and Tao Zhou

Last updated by author(s): Sep 11, 2023

Reporting Summary

Nature Portfolio wishes to improve the reproducibility of the work that we publish. This form provides structure for consistency and transparency in reporting. For further information on Nature Portfolio policies, see our Editorial Policies and the Editorial Policy Checklist.

Statistics

For all statistical analyses, confirm that the following items are present in the figure legend, table legend, main text, or Methods section.

n/a	Confirmed	
☐	☒	The exact sample size (n) for each experimental group/condition, given as a discrete number and unit of measurement
☐	☒	A statement on whether measurements were taken from distinct samples or whether the same sample was measured repeatedly
☒	☐	The statistical test(s) used AND whether they are one- or two-sided<br>*Only common tests should be described solely by name; describe more complex techniques in the Methods section.*
☒	☐	A description of all covariates tested
☐	☒	A description of any assumptions or corrections, such as tests of normality and adjustment for multiple comparisons
☒	☐	A full description of the statistical parameters including central tendency (e.g. means) or other basic estimates (e.g. regression coefficient) AND variation (e.g. standard deviation) or associated estimates of uncertainty (e.g. confidence intervals)
☒	☐	For null hypothesis testing, the test statistic (e.g. F, t, r) with confidence intervals, effect sizes, degrees of freedom and P value noted<br>*Give P values as exact values whenever suitable.*
☒	☐	For Bayesian analysis, information on the choice of priors and Markov chain Monte Carlo settings
☐	☒	For hierarchical and complex designs, identification of the appropriate level for tests and full reporting of outcomes
☒	☐	Estimates of effect sizes (e.g. Cohen's d, Pearson's r), indicating how they were calculated

Our web collection on statistics for biologists contains articles on many of the points above.

Software and code

Policy information about availability of computer code

Data collection	Basecalling of nanopore raw reads were performed by Guppy (version 3.1.0).
Data analysis	De novo genome assembly was performed with NextDenovo (https://github.com/Nextomics/NextDenovo, version 2.5.0).<br>Genome completeness was evaluated with BUSCO (version 3.1.0) and CEGMA (v2).<br>Genome sequence comparison was performed with LASTZ (version 1.04.15), short sequence alignments were performed with bowtie2 (version 2.4.5), long sequences alignments were performed with minimap2 (version 2.24-r1122) and multiple sequence alignment was performed with Clustal Omega (version 1.2.4).<br>Short tandem repeats were identified with TRF (version 4.09.1) and transposon-based elements were identified with RepeatMasker (version 4.1.2).<br>Haplotype assemblies were performed with Canu (version 2.2).<br>Immune repertories were profiled by MiXCR (version 4.1)<br>Sankey diagram were performed with SankeyMATIC software (https://sankeymatic.com).<br>Phylogenetic analyse was visualized using the Interactive Tree of Life software (version 6.6).<br>V-(D)-J gene clusters were manually annotated following the IMGT criteria with SnapGene (version 6.1.2), and visualization were carried out with in-house R script (version 4.2.1).<br>The interproscan GO database can be publicly accessible at https://github.com/ebi-pf-team/interproscan.<br>The KEGG Database can be publicly accessible at https://www.genome.jp/kegg/.<br>The KOG Database can be publicly accessible at https://www.ncbi.nlm.nih.gov/research/cog.<br>The NR Database can be publicly accessible at https://ftp.ncbi.nlm.nih.gov/blast/db/.<br>The Swiss-Prot Database can be publicly accessible at https://www.expasy.org/resources/uniprotkb-swiss-prot.

The related codes and figures for reproducible research are stored at GitHub (https://github.com/TintingLi/cattleGenome).

For manuscripts utilizing custom algorithms or software that are central to the research but not yet described in published literature, software must be made available to editors and reviewers. We strongly encourage code deposition in a community repository (e.g. GitHub). See the Nature Portfolio guidelines for submitting code & software for further information.

Data

Policy information about availability of data

All manuscripts must include a data availability statement. This statement should provide the following information, where applicable:

- Accession codes, unique identifiers, or web links for publicly available datasets
- A description of any restrictions on data availability
- For clinical datasets or third party data, please ensure that the statement adheres to our policy

The raw sequence data reported in this paper have been deposited in the Genome Sequence Archive in National Genomics Data Center, China National Center for Bioinformation under accession number CRA006888 (https://bigd.big.ac.cn/gsa/browse/CRA006888) that can be publicly accessible at https://ngdc.cncb.ac.cn/gsa. The whole genome assembly and annotation data of NCBA_BosT1.0 have been deposited in the Genome Warehouse in National Genomics Data Center under accession number GWHBISA00000000 (https://ngdc.cncb.ac.cn/gwh/Assembly/25200/show) that can be publicly accessible at https://ngdc.cncb.ac.cn/gwh. The related codes and figures for reproducible research are stored at GitHub (https://github.com/TintingLi/cattleGenome) and Zenodo (https://doi.org/10.5281/zenodo.8334734).

Human research participants

Policy information about studies involving human research participants and Sex and Gender in Research.

Reporting on sex and gender	N/A
Population characteristics	N/A
Recruitment	N/A
Ethics oversight	N/A

Note that full information on the approval of the study protocol must also be provided in the manuscript.

Field-specific reporting

Please select the one below that is the best fit for your research. If you are not sure, read the appropriate sections before making your selection.

☒ Life sciences ☐ Behavioural & social sciences ☐ Ecological, evolutionary & environmental sciences

For a reference copy of the document with all sections, see nature.com/documents/nr-reporting-summary-flat.pdf

Life sciences study design

All studies must disclose on these points even when the disclosure is negative.

Sample size	The genome assembly was performed on an individual female cattle, and thus sample size was not considered in the study design.
Data exclusions	No data were excluded from the analyses.
Replication	All experiments were replicated or performed independently, and all attempts at replication were successful.
Randomization	The genome assembly was performed on an individual female cattle, and thus randomization was not relevant to our study.
Blinding	The investigators were blinded to group allocation during data collection and/or analysis.

Reporting for specific materials, systems and methods

We require information from authors about some types of materials, experimental systems and methods used in many studies. Here, indicate whether each material, system or method listed is relevant to your study. If you are not sure if a list item applies to your research, read the appropriate section before selecting a response.

Materials & experimental systems

n/a	Involved in the study	
x		Antibodies
x		Eukaryotic cell lines
x		Palaeontology and archaeology
	x	Animals and other organisms
x		Clinical data
x		Dual use research of concern

Methods

n/a	Involved in the study	
x		ChIP-seq
x		Flow cytometry
x		MRI-based neuroimaging

Animals and other research organisms

Policy information about studies involving animals; ARRIVE guidelines recommended for reporting animal research, and Sex and Gender in Research

Laboratory animals	Holstein cattle, one year old.
Wild animals	No wild animals were used in the study.
Reporting on sex	Female.
Field-collected samples	No field-collected samples were used in the study.
Ethics oversight	The procedures were performed in strict accordance with the Guide for the Care and Use of Laboratory Animals. All the animal work in this study was approved by the ethics committee of China Agricultural University (No. 2017-04-11).

Note that full information on the approval of the study protocol must also be provided in the manuscript.
